# A Cross-Media Advertising Design and Communication Model Based on Feature Subspace Learning

**DOI:** 10.1155/2022/5874722

**Published:** 2022-05-17

**Authors:** Shanshan Li

**Affiliations:** City College of Dongguan, Dongguan 523104, China

## Abstract

This paper uses feature subspace learning and cross-media retrieval analysis to construct an advertising design and communication model. To address the problems of the traditional feature subspace learning model and make the samples effectively maintain their local structure and discriminative properties after projection into the feature space, this paper proposes a discriminative feature subspace learning model based on Low-Rank Representation (LRR), which explores the local structure of samples through Low-Rank Representation and uses the representation coefficients as similarity constraints of samples in the projection space so that the projection subspace can better maintain the local nearest-neighbor relationship of samples. Based on the common subspace learning, this paper uses the extreme learning machine method to improve the cross-modal retrieval accuracy, mining deeper data features and maximizing the correlation between different modalities, so that the learned shared subspace is more discriminative; meanwhile, it proposes realizing cross-modal retrieval by the deep convolutional generative adversarial network, using unlabeled samples to further explore the correlation of different modal data and improve the cross-modal performance. The clustering quality of images and audios is corrected in the feature subspace obtained by dimensionality reduction through an optimization algorithm based on similarity transfer. Three active learning strategies are designed to calculate the conditional probability of unannotated samples around user-annotated samples in the correlation feedback process, thus improving the efficiency of cross-media retrieval in the case of limited feedback samples. The experimental results show that the method accurately measures the cross-media relevance and effectively achieves mutual retrieval between image and audio data. Through the study of cross-media advertising design and communication models based on feature subspace learning, it is of positive significance to advance commercial advertising design by guiding designers and artists to better utilize digital media technology for artistic design activities at the level of theoretical research and applied practice.

## 1. Introduction

Based on computer and network information technology, digital media technology is an interdisciplinary and comprehensive application of theoretical knowledge from multiple disciplines such as design, psychology, and optics. Since its creation, it has influenced the design field with its diversified artistic creation and expression and has been widely used in many art and design fields such as urban planning, TV and film production, exhibition and trade show, and commercial advertising design [1]. It can be said that the addition of digital media technology has added more diversified artistic elements and expressions to the artistic presentation of these fields. Digital media technology has a strong technological advantage and visual expression. Through the full mobilization of the user's senses, it break through the limitations of the traditional two-dimensional static image technology and form a multidimensional information presentation form. From simple visual perception to multisensory experience of vision, hearing, and touch, the user can change from the passive receiver to the information presenters and disseminators [2]. At the same time, the full media integration of digital media technology makes all kinds of information which need to be disseminated from an online and offline network communication mode, so that information access becomes available at your fingertips. Its diversified creation methods and innovations in visual culture communication have added more creative artistic elements and artistic expressions to art design, which play an important role in attracting users to watch, delighting their emotions, evoking their emotional resonance, and promoting the production of several excellent art design works [3].

With the advent of the Internet era, a large amount of digital information is requested and transmitted every day. These digital messages have different modalities such as text, images, video, and audio. Not only are the data on the Internet huge and modally diverse, including images, text, audio, and video, but also they usually appear in pairs and are semantically interrelated, such as an illustrated article. Using data of different modalities to describe the same thing can show all aspects of the thing more intuitively and help people understand the thing better. In the era of big data, how to get valuable information from huge and complicated multimodal data is a problem worth focusing on [4]. At present, unimodal retrieval techniques such as image retrieval and text retrieval have been widely studied, but these techniques can only return the same retrieval results as the query sample modality. In contrast, cross-modal retrieval is more convenient and flexible, which can complete information retrieval across different modalities flexibly by mining the association information between pairs of cooccurring cross-modal data; that is, it can return the retrieval results of other modalities related to the query sample according to the sample of any query modality.

Along with the continuous development of interactive technology, the multiplication of Internet technology, and the deep application of digital technology, various new media methods have emerged [5]. Compared with traditional media, which are mature, stable, and easy to implement, new media emphasize the content and form of multisensory interactive experience, which further broadens the connotation and extension of visual communication design. Nowadays, the trend is to integrate the advantages of traditional media and new media, and it is imperative to take “cross-media” as the focus to explore a new path for the visual and communication innovation of PSAs, which is in line with the public evaluation in the contemporary context [6]. Transmedia can give PSAs more creative space and stronger technical support in terms of content, form, and communication. The use of cross-media can greatly enrich the visual presentation of PSAs, strengthen the multisensory interactive experience with audiences, deepen the content expression of public service themes, and enhance the efficiency of public service issues and participation.

## 2. Related Works

Subspace learning methods use pairwise cooccurrence information of different modal sample pairs to learn a common subspace and measure the similarity of different modal data in the subspace. Typical correlation analysis (CCA) has been covered in several studies as an early classical method [7]. Ahmed et al. proposed a two-stage method for cross-modal multimedia retrieval of Wan. In the first stage, CCA learns a subspace by maximizing the correlation between two modalities and then learns a semantic space by logistic regression to measure the similarity between different modal features [8]. Liang et al. introduced multilabel canonical correlation analysis (ml-CCA), which is an extension of CCA to learn a shared subspace by considering high-level semantic information in the form of multilabel annotations [9]. A fast ml-CCA, a computationally efficient version of ml-CCA capable of handling large-scale datasets, is also provided. These methods are well trained and easy to implement as classical methods in cross-modal retrieval subspace learning, but they lack in-depth study of the semantic structural correlation between modalities. Liu et al. proposed a new ranked classical correlation analysis (RCCA) for learning query and image similarity [10]. The subspace of CCA learning is adjusted using RCCA to further maintain the preference relations in click data. Meanwhile, Das et al. proposed a supervised extension of CCA called generalized multiview analysis (GMA). It extends linear discriminant analysis (LDA) and Marginal Fisher Analysis (MFA) to their contained extensions to their multiview counterparts and applies them to the cross-media retrieval problem [11].

Bouet et al. found in their study of brand-consumer buying behavior in the emerging field of computational advertising that technological advances and technology have made it possible for companies to understand their target audiences and better design their commercials [12]. Kaur discusses two ways in which art design can help people receive and understand information. In a study by Heejun Lee et al. on the status and outlook of digital media technology applications, it is suggested that new digital technologies have greatly changed the way companies communicate and interact with consumers and that data-driven marketing communications, artificial intelligence, and big data technologies have had a significant impact on the design, production, and promotion of commercial advertising [13]. Ilango et al. analyzed the main factors influencing the design and communication effects of social network-based commercials from the perspective of users' cognitive behaviors, including the value of advertising, users' reception of advertising, and individual users' behavioral and cognitive factors [14]. Al-Faramawi et al. discuss the two-way integration of advertising and design from a cross-media perspective and propose that, in the digital media era, traditional commercial advertising design methods can no longer meet the demand of “everything is a medium,” and commercial advertising design faces a new challenge of achieving cross-media communication based on art, technology, and content [15].

In summary, the theoretical research on the application of digital media technology in commercial advertising design has formed certain milestones, but the research results on the impact mechanism and effect of digital media technology on commercial advertising design are still insufficient. Some of the research results have discussed the impact of technological development on commercial advertising design from digital media technology; some of the research results have studied the changes of users' visual feelings and aesthetic values from users' feelings of use [16]. There are fewer studies that combine the characteristics of digital media technology and the development history of commercial advertising design and explore in depth the mechanism and influence relationship between them. This paper intends to explore the important influence of digital media technology on commercial advertising design from the perspective of digital media and the communication characteristics of commercial advertising design, combined with the analysis of successful cases of commercial advertising design using digital media technology, and also explore the important influence of digital media technology on commercial advertising design from the perspective of digital media, design content, and design thinking to promote commercial advertising design to be closer to the current development trend of art and technology in the new media era.

## 3. Cross-Media Model Design for Feature Subspace Learning

From the existing feature subspace learning models, most forms of constraint terms have contributed to the learning of feature subspaces. Since its introduction, the Low-Rank Representation model has been widely studied in the direction of subspace learning, subspace clustering, and image processing, and it can better discover the low-dimensional structural composition of sample data accompanied by interference suppression. Therefore, in this paper, we design a feature subspace constraint term based on Low-Rank Representation and sample correlation. This constraint term integrates the Low-Rank Representation with the feature space, explores the potential essential structure of the sample using the Low-Rank Representation model, and constrains the nearest neighbor of the sample in the projection space by using the Low-Rank Representation coefficients as a metric of the potential essential structure. COIL20 is an object dataset containing 1440 images from 20 objects, and each object contains 72 images taken continuously at an angle of 5°. In the experiments, all images in the dataset are cropped to 32 × 32 pixels, and 10 images from each object image are randomly selected as the training set, and the remaining images are used as the test set.

(1) $$\begin{array}{c} S_{ij}={\left\|X_{ij}P^{T\left(K+1\right)}\right\|}^{2}, \end{array}$$

(2) $$\begin{array}{c} \underset{P,Z}{\text{Max}}\sum_{ij=1}Z_{ij}{\left\|P^{T}X_{i}-P^{T}X_{j}\right\|}^{2}. \end{array}$$

**Figure 1:**
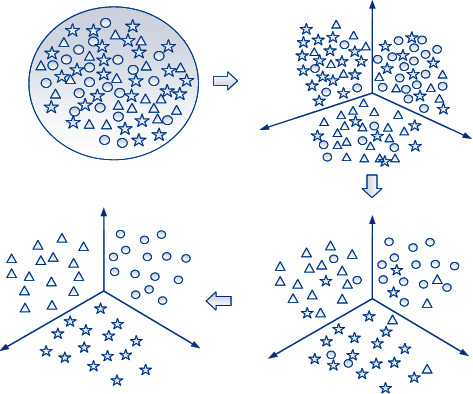
Schematic diagram of the effect of robust feature learning model with Low-Rank Representation constraint.

*Z*_*ij*_ can be used to measure the structural similarity between samples *X*_*i*_ and *X*_j_ to maintain the local structure of the samples. In this paper, an additional nonnegative constraint is imposed on *Z* to ensure the nonnegativity of the distance constraint term. If the similarity between two samples is greater, *Z*_*ij*_ is larger, and vice versa. In addition, to reduce data redundancy, an additional orthogonality constraint is introduced here on the unit array of the projection matrix P *P*^*T*^*P*=*I*_*p*_ × *p*. The schematic diagram of the projection effect of the model in equation (2) is shown in Figure 1. Among them, the triangles, squares, and circles of different colors represent five different categories of samples, respectively. To further enhance the discriminative and robustness of the feature subspace, this paper proposes a constraint term for the feature subspace based on low-rank reconstructed samples. The constraint term first uses Low-Rank Representation coefficients for sample reconstruction, which aims to suppress noise interference and recover pure original data to a certain extent, and then uses label information to constrain the distance relationship of samples in the feature projection space in a supervised manner.

With the rapid development of information science and technology, big data has become an important basic resource for the development of countries, enterprises, and individuals. At the same time, an important feature of data has come into being, high dimensionality [17]. The subspace clustering algorithm can well learn the hidden low-dimensional features under the high-dimensional data so that the structural features of the data can be obtained more rapidly and effectively. Introduce what is subspace clustering from the mathematical level. Let a given dataset *X*={*x*_*i*_ ∈ *P*^*d*^}, denote that there are *n* data points in the dataset, and these data sample points are obtained from the concatenation of *K* unknown linear subspaces {*S*_1_, *S*_2_,…, *S*_*k*_}, each with dimension {*d*_1_, *d*_2_,…, *d*_*k*_} and base *B*_1_ ∈ *R*^*d*_1_^, *B*_2_ ∈ *R*^*d*_2_^, *B*_*k*_ ∈ *R*^*d*_*k*_^. The purpose of subspace clustering is to divide the data into *K* different feature subsets based on their characteristics, such as *X*=*X*_1_+*X*_2_+⋯+*X*_*k*_, and *X*_*i*_ is the set of sample data points from subspace *S*_*i*_.

(3) $$\begin{array}{c} f\left(Z\right)=\text{min}f\left(x\right)+ag\left(Z\right)+ag\left(E\right),\\ Z=\underset{i=1}{\text{Max}}\sum_{j=1}\left|Z_{ij}\right|. \end{array}$$

Typical correlation analysis CCA is easy to implement, but it can only mine the linear projection correlation between different modal data, and when the relationship between multiple modal data is not simple enough, this method usually causes semantic information loss [18]. To make full use of the semantic information, some scholars have introduced nonlinear projection methods into CCA methods, which can be broadly divided into the two following types, namely, fusion deep learning methods and adding kernel functions. The first method is the deep typical correlation analysis DCCA proposed by Andrew et al. The deep typical correlation analysis method has excellent nonlinear feature mapping capability and can output the maximum correlation by learning the parametric nonlinear transformation of two random vectors. In the specific implementation, the data of two modalities are linearly correlated by complex nonlinear transformations through a neural network. The network structure of DCCA is illustrated, which is divided into two neural networks, and the output layer (topmost layer of each network) with the maximum correlation is obtained by training. Specifically, two neural networks *f* and *g* are used to extract the nonlinear features of the two types of data, respectively. Any intermediate layer in each view network has 4 units, the output layer has B units, and the output layer (*B* = 2) has the maximum correlation. The nodes in the bottom layer corresponding to the input features (*n*_1_=*n*_2_=2) and the nodes in the three middle layers (*a*_1_=*a*_2_=5) all represent hidden units.

(4) $$\begin{array}{c} \text{s,t,}U<0,\;V\leq 0. \end{array}$$

**Figure 2:**
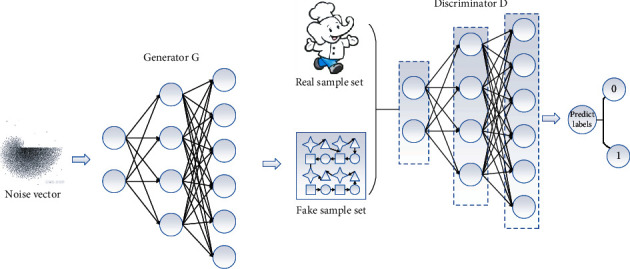
Schematic diagram of the GAN framework.

Generative adversarial networks (GANs) have become one of the widely researched hotspots in recent years since they were proposed by Goodfellow et al. in 2014, and many algorithms related to them have been proposed frequently, especially in the field of computer vision. Inspired by game theory and the two-person zero ideas, GAN is a deep learning model that uses adversarial learning to estimate the generative data, aiming to learn the distribution of real data (training set data). The framework structure of GAN is shown in Figure 2 and consists of two parts: a generator (*G*) and a discriminator (*D*). *g* focuses on generating fake data close to the real data distribution based on the input sample data, and *d* focuses on discriminating the real data from the fake data in the input data. Both *G* and *D* are functions by nature and are usually chosen for deep neural network implementation. Let *G*(*z*) denote the output generated data, *P*(*z*) denote the distribution of the generated data, and *P* (*x*) denote the distribution of the true samples, with *G* maximizing *P*(*z*) to be equivalent to *P* (*x*) as much as possible. *d* focuses on discriminating the most accurate data possible from the received main sentence, and its input consists of the true data *X* and the generated data *G*(*z*). *D* ultimately outputs a probability value that represents the probability that the received input is true. The probability output by *D* will be returned to *G*, and the two will undergo uninterrupted adversarial training until finally *G* learns the distribution of the real data so that *D* cannot judge the truth or falsity of the received data, in which case we decide that *G* has learned the distribution of the real data and the model is optimal with better output.

The GAN is trained by an iterative numerical method, and, to prevent overfitting, the optimization of *D* is usually performed *k* times before the optimization of *G*. This can ensure that the learning ability of *G* and *D* can be improved simultaneously during the training process. With this alternate training approach, the problem of inconvenient operation of simultaneous model training can be solved. As mentioned above, the discriminator network is essentially dichotomized, so the real samples are marked with 0 and 1 in advance to facilitate training. The discriminator network is essentially dichotomized, so the real samples and the fake datasets are marked in advance for training purposes, with 1 marking the real samples and 0 marking the generated fake data. With the help of the discriminator, the generator learns the real sample distribution and generates as realistic samples as possible to deceive the discriminator.

## 4. Experimental Designs for Advertising Design and Communication Applications

The ability of film and television advertising to be an expression is related to symbols. Pierce divides symbols into three kinds of symbols: image symbols, sign symbols, and symbols, among which image symbols are like its referents, the most common being graphics, but they can also be sounds, smells, and so forth. The creativity of film and television advertising is to seek change, wonder, and novelty in the form of symbols; in particular, the presentation of mainly image symbols can maximize its influence in terms of breadth and depth. Among them, the use of graphic design symbolic principles in film and television advertising is concentrated in the color, graphics, text elements, and their innovation [19]. Color symbols are an important part of graphic design and an indispensable visual symbol element in film and television advertising. People's feelings about color come from the interactive unity of form and content. First, the use of reasonable senses directly feels the stimulation of material color and the resulting formation of color perception feelings; second, use the level of emotional cognition to establish association habits and thinking structure to have a deeper understanding of the perception of color alternation. The rational use of color is the enrichment of the language of film and television expression, and, with the growth of video picture postproduction technology, the color formed in the photo can also be changed as well as second-degree decoration to achieve the results that the creative person wants to get, so it is also more able to add the charm of the use of artworks. Not only are the data on the Internet huge in scale and diverse in modalities, including pictures, texts, audio, and video, but also they usually appear in pairs and are semantically related, such as an article with pictures and texts.

In practice, the adversarial learning between the two is achieved by optimizing the parameters, which requires the generated data to be labeled as 1 in advance to deceive the discriminator, at which time the discriminator will generate a large “doubt,” i.e., error, due to the false sample and pass it back to the generator and adjust it accordingly; if the generated data are close to the real sample distribution and the label is also 1, the discriminator generates less error. Through these two processes, adversarial learning is achieved, and the generated forged data approximates the true sample distribution, and the discriminative network cannot distinguish between the true and false accepted data. It is important to clarify that the training parameters of the generator are changed only when the parameters are passed back to the generator to achieve the optimization process. One of the characteristics of GANs, namely, the alternation of true and false samples in training, is also the focus in adversarial learning advancement. Generative adversarial networks (GANs) can be applied to a wide range of modal data such as images, text, and video in specific applications and can likewise be applied to a variety of research tasks such as image generation, text generation, style migration, and video screen prediction.

Like the MOAR algorithm, the basic idea of the MCAR algorithm is to capture the time-domain correlation between successive tensor time slices using an AR model within the framework of the Tucker decomposition. Although the local geometric structure information and time-domain dependence of the original time series can be well preserved in the form of tensor, the inherent distortion, missing, and redundancy in the data are still unavoidable problems in time series analysis [20]. Based on the MOAR model, we add an inverse decomposition error constraint term, whose core idea is to minimize the error between the observed kernel tensor time series slices and the reconstructed kernel tensor time series slices. In the field of pattern analysis and signal processing, data usually contain some underlying structural information. Mining these can make the presentation and processing of data more efficient. Low-rank subspace learning is a commonly used and representative structural assumption. For example, the classical Robust Sparse Principal Component Analysis (RSPCA) and Low-Rank Representation (LRR) are widely used in dimensional simplification, data recovery, classification, prediction, and other tasks, which woodenly assume that the number batches are distributed in a low-autumn subspatial structure. In the experiments, all images in the dataset are cropped to 32 × 32 pixels, and 10 images are randomly selected from each object image as the training set, and the remaining images are used as the test set.

(5) $$\begin{array}{c} \left\|A\right\|=\text{Rank}\left(A\right)+\lambda {\left\|E\right\|}_{0}^{2},\\ {\left\|A\right\|}^{'}=\sum_{t=1}^{p}\delta_{t}\left(A\right). \end{array}$$

**Figure 3:**
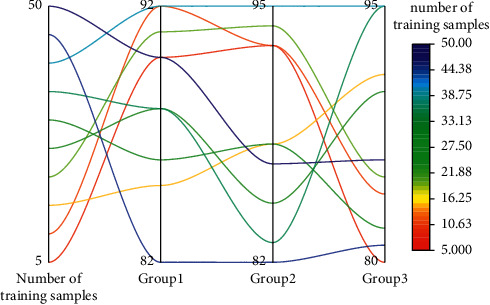
Percentage of noise classification.

Although the classical LRR has achieved better results as an effective algorithm in exploring the subspace structure of data, such as subspace clustering and matrix recovery, LRR directly uses the original observed data matrix as a dictionary to mine the potential structural information of the data. However, one of the drawbacks of this processing method is that when the sample data is not sufficient, it causes the derived sample Low-Rank Representation matrix to be not sufficient and robust. In this context, Liu et al. then proposed the Latent Low-Rank Representation (LatLRR) algorithm. Given a set of *N* lossy sample data consisting of a matrix *X*=[*X*_1_+*X*_2_+...*X*_*N*_] ∈ *R*^*D*×*N*^, where *X*_*I*_ ∈ *R*^*D*^, *D* represents the feature dimension of the samples. LatLRR assumes that sample reconstruction recovery requires the existing observed samples and is also related to the hidden set of observed samples, as shown in Figure 3.

The all-media integration of digital media technology enables various types of information to be disseminated to form an online and offline network dissemination mode, making information acquisition within easy reach. This subsection introduces a classical algorithm for subspace clustering, Sparse Subspace Clustering (SSC), which introduces a compression-aware technique to perform subspace partitioning to learn the sparsest representation of the data; notably, the sparse representation matrix here is block sparse, making the data within classes sparse (represented by nonzero data), and the interclass data are all represented by zeros. It means that there are as many subspaces as there are block matrices in the matrix, and the dimensionality of the block matrix indicates the dimensionality of each subspace. Thus, it is possible to distinguish different classes of data very effectively and achieve the effect of subspace partitioning. From the introduction of the subspace clustering algorithm in Chapter 1, we know that the traditional subspace clustering algorithm needs to know the number of subspaces and the dimension of each subspace in advance, while SSC does not need to know the dimension of each subspace and theoretically does not need to know the number of subspaces in advance, does not need to be set up initially, can handle data points at the boundary of subspace, and can handle data noise, outliers, and missing data. The SSC algorithm is the first algorithm that directly uses the subspace boundaries. The SSC algorithm is the first to directly use the sparse representation of vectors distributed over subspaces and sets to aggregate data into separate subspaces. SCC has a prerequisite that each data on a subspace is assumed to be representable as a linear combination of all other data points.

**Figure 4:**
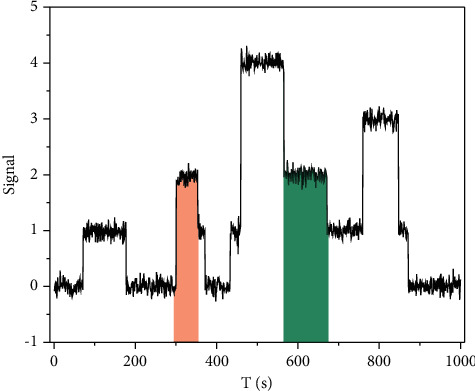
Verification test of LRR algorithm's ability to automatically correct errors in data.

In real-life applications, the data we obtain is often not complete; there will be data loss and corruption, so if we learn the representation matrix of clean and uncontaminated data only from the original data, its accuracy is very low, and it does not match the characteristics of the data obtained in real life, which urgently requires us to have the ability to separate clean and noisy data from the original data, which can greatly enhance the algorithm's robustness; the LRR algorithm has a strong ability to correct the wrong information in the data, and the verification is given in Figure 4, which is given in the original paper, where (a) is the contaminated data, (b) is the representation matrix learned by the model, and (c) is the clean data after being processed; the representation matrix *Z* is the shape of a diagonal block, and the clean data learned by the model is essentially free of noise. Similarly, the robustness of LRR is further demonstrated by the ability to recover the correct data very accurately in the case of corrupted data.

## 5. Performance Analysis of Cross-Media Models with Feature Subspace Learning

The classical algorithm Sparse Subspace Clustering (SSC) introduces a compression-aware technique for subspace partitioning, trying to learn the sparsest representation of the data; it is worth noting that the sparse representation matrix here is block sparse so that the data within the class is sparse (represented by nonzero data) and the data between the classes are all represented by 0. The dimensionality of the block matrix represents the dimensionality of each subspace. Thus, it is possible to distinguish different classes of data very effectively and achieve the effect of subspace partitioning. From the introduction of the subspace clustering algorithm in Chapter 1, we know that the traditional subspace clustering algorithm needs to know the number of subspaces and the dimension of each subspace in advance, while SSC does not need to know the dimension of each subspace and theoretically does not need to know the number of subspaces in advance, does not need to be set up initially, can handle data points at the boundary of subspace, and can handle data noise, outliers, and missing data. Data from the media technology transforms pure visual perception into visual, auditory, and tactile multisensory experience, enabling users to transform from passive recipients of pure information to participants in information display and dissemination. It can handle data points at the boundary of subspace, data noise, outliers, and missing data.

To verify the effectiveness of each part of the method in this chapter, targeted ablation experiments were designed, in which “no linear discriminant” means removing the linear discriminant analysis term from the total objective function, “no graph regularization” means removing the graph regularization term from the total objective function, and “non-task-oriented” means combining the unimodal semantic regression term into multimodal semantic regression and the unimodal linear discriminant analysis term into the multimodal linear discriminant analysis; that is, the same objective function is used for different retrieval tasks. The experimental results show that intraclass aggregation and interclass distancing of the samples of retrieval modality by linear discriminant analysis can effectively ensure their semantic consistency. This item performs better in the I2T task because the text features are more discriminative. Constructing KNN local nearest-neighbor graphs for data of different modalities can better preserve the intramodal similarity. Ignoring the variability of different tasks, using multimodal semantic regression and multimodal linear discriminant analysis, learning only one set of mappings instead prevents the performance in each task from reaching the respective optimum. In addition, the linear discriminant analysis term and the graph regularization term can contribute to each other to further improve the accuracy of cross-modal retrieval.

**Figure 5:**
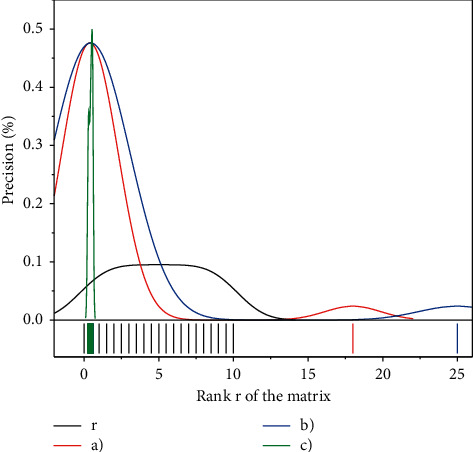
Relationship between the matrix rank *r* and the check rate.

The singular value decomposition of the cross-media feature coeval matrix *E* yields the approximation matrix *E*^*∗*^ of *E* of rank *r* in the least-squares sense. However, the problem is that there is no theory on exactly how many dimensions should be retained in the obtained subspace to minimize noise while retaining more complete cross-media data relationships. The effect of changing *r* values on cross-media retrieval performance in the experiment is given in Figure 5. In Figure 5, the accuracy rate is the average of the accuracy rate obtained when the number of returned results is 15 for both image retrieval with audio and audio retrieval with image, without relevant feedback. When *r* = 9, although more noise is removed, the retained cross-media data relationships also become incomplete, so the retrieval performance is low. *r* is in the range of [21, 24] when the search accuracy is higher, close to 0.45.

**Figure 6:**
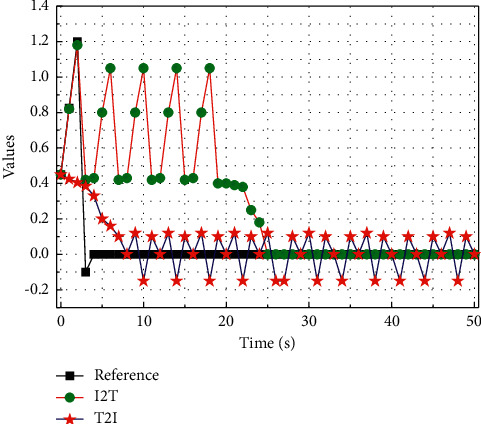
Comparison results of model convergence curves.

In the Wikipedia dataset, the graph regularization term has a large impact on the training time, and the linear discriminant analysis term has almost no impact on the training time; meanwhile, in the Pascal Sentence dataset, adding either graph regularization or linear discriminant analysis can reduce the training time. The main reason is that the semantic categories of the Wikipedia dataset are very abstract, and the differences between the categories of the same modal data are not obvious, which leads to the slow convergence after adding graph regularization, while the categories of the Pascal Sentence dataset are obvious, and adding these two terms helps convergence. In addition, comparing the training times of the non-task-oriented method and this chapter's method, the convergence speed of different retrieval tasks in the task-oriented method is different, but the average training time is not much different compared with the non-task-oriented method. The convergence curves plotted on the two datasets are shown in Figure 6. The results show that the objective function values of this chapter's method gradually stabilize with the increase of the number of iterations and generally converge after 5 iterations.

In this chapter, a task-oriented cross-modal retrieval method with joint linear discriminant and graph regularity is proposed with different mapping mechanisms for different retrieval tasks. The correlation between different modal pairwise samples and the regression of query modal sample features to their corresponding semantic vectors are combined to map multimodal data to a common subspace, while the semantic consistency of retrieval modal samples is fully ensured by using the linear discriminant analysis term of retrieval modal in the joint learning process, and the local nearest-neighbor graph is constructed for each modal to mine the original distribution information within its modalities, to obtain more data matching information. The experimental results are compared with 12 existing methods on two cross-modal datasets and they fully demonstrate the effectiveness of the method in this chapter. In addition, the objective function of this chapter is not limited to a specific modality but can be easily extended to other modalities besides image text and can be superimposed on any modality; thus it can theoretically achieve the retrieval of more than two modalities in the same framework.

## 6. Analysis of Application Results

**Figure 7:**
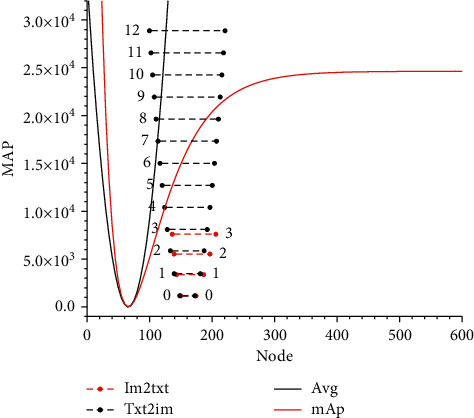
mAP values of different hidden nodes *W*.

Given the graph *G*=*g*(*V*, *E*), *V* denotes the set of nodes in the community network, and *E* denotes the set of relationships between the edges of the nodes of the community network; we roughly divide the community detection algorithm based on the subspace representation into three steps, and the overall flow of the subspace-based community detection algorithm is given in Figure 7. The adjacency matrix *A* of graph *G* is known, and, in the first step, the shortest path between each node is calculated on the adjacency matrix *A* using the shortest path solving method to obtain the geodesic distance matrix *P*. Next, the geodesic distance matrix is optimized and converted into the similarity matrix *S*. The similarity matrix *S* not only reflects the topological map between the nodes of the actual community network but also describes the similarity between the nodes so that the nodes have high similarity. In the second step, *S* is used as data input and applied to the correlation clustering model of subspace representation to learn the low-dimensional data structure of the network; for example, SSCF and LRSCD algorithms are to learn the sparse and Low-Rank Representation of the data, respectively; in the third step, the subspace representation matrix and the adjacency matrix *A* are fused and then clustered to divide the communities in the network.

Parameter *W* indicates the number of different hidden nodes, and Figure 7 shows the mAP scores of different *W* corresponding to the retrieval task. The hidden nodes between 1 and 200 were selected for comparison after screening. From the experimental results, in the average mAP of the graphical interrogation, the mapping score is highest when the number of hidden nodes *W* is 19. Before that, with the increase in the number of hidden nodes, the three curves in Figure 7 increase sharply and then decrease slowly but continuously, indicating that this node is the corresponding hidden node when the retrieval performance is the best, so parameter *W* is set to 19 to continue the subsequent comparison experiments.

**Figure 8:**
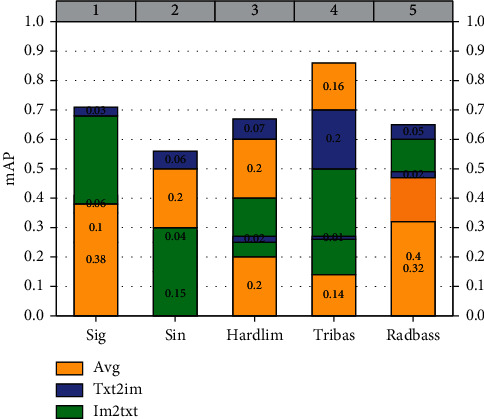
mAP values of different activation functions.

After determining the optimal number of hidden nodes, we conducted comparison experiments for different activation functions, and the experimental results are shown in Figure 8. The experiments show that the mAP scores of the activation function “sig” are the highest compared with other activation functions, reaching 0.5234, 0.439, and 0.1685 for the tasks of image retrieval text and text retrieval image as well as the average mAP, so “sig” is chosen as the activation function in the subsequent experiments. Therefore, “sig” was chosen as the activation function in the subsequent experiments.

**Figure 9:**
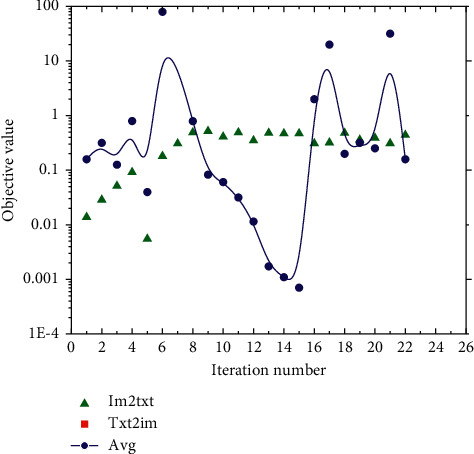
Trend of loss function of the dataset.

We analyze the convergence and calculate the mean score values of their prediction results. On the other hand, we also calculate the RC and *R* values in the predicted results and the true results. To verify the convergence of the feature subspace algorithm, we test the convergence of the objective function based on the proposed iterative solution algorithm.  Figure 9 shows the relationship between the number of iterations and convergence, where the *x*-axis represents the number of iterations and the *y*-axis represents the relative change of the objective function between two consecutive iterations. From this figure, the difference between the objective functions decreases rapidly with the increase in the number of iterations. When the number of iterations exceeds 20, the algorithm almost converges. Therefore, we set the convergence condition for the algorithm that the relative change of the objective function value between two consecutive iterations does not exceed 0.1 and the maximum number of iterations does not exceed 20.

Robust subspace learning with adaptive parameters is extended to the multiview algorithm model. In the multiview subspace clustering algorithm MVSC proposed in this paper, the parameters of each viewpoint can be learned adaptively from its viewpoint data, which neither misses the best parameters by setting uniform parameter values nor makes the model waste a lot of time in debugging parameters by traversing to set all possible parameter values, and the parameters learned from the data itself to balance the weights of each viewpoint data sample are often optimal. The experimental results also confirm that the MVSC algorithm outperforms all other comparative methods. Combining subspace learning with adaptive parameter setting and community networks, the adjacency matrix of the network is first mapped to the geodesic space to obtain another representation of the data, that is, the geodesic distance matrix, and then the Gaussian similarity matrix obtained from the geodesic distance matrix, followed by applying a subspace clustering algorithm to learn the potential data subspace structure in the similarity matrix. The algorithm learns the global and local stream structure of the network data, is robust to noise in the data, and still has a strong learning ability for networks with ambiguous community structures. Experiments on both synthetic and real datasets validate the excellent performance of the algorithm.

## 7. Conclusion

The paper presents a discriminative feature subspace learning method for image feature extraction. The method incorporates Low-Rank Representation and subspace learning into the same framework. Using the Low-Rank Representation coefficients as the similarity constraint of samples in the projection space, the distance constraint term of samples in the feature space is constructed so that the projection subspace can better maintain the local nearest-neighbor relationship of samples; a discriminative feature space constraint term is designed using low-rank reconstruction of training samples to improve the noise resistance of the model; a label regression-based feature space constraint term is introduced to effectively enhance the discriminative feature space and interclass discriminative adaptation capability. To meet the retrieval needs of users, we wish to design a graphical cross-modal retrieval system in which the DCCA-ACMR method with excellent cross-modal retrieval performance is applied. To better guide the system implementation, the system requirements were fully analyzed in advance, based on which a system based on the B/S framework was designed, considering that the core module of the system is the cross-modal retrieval function module, focusing on the realization of feature conversion and similarity calculation in it, and finally the user-system interaction was realized with the help of visual operation interface to realize the tasks of image retrieval of text and text retrieval of image.

## Data Availability

The data used to support the findings of this study are available from the corresponding author upon request.
